# Letters and biological process: linguistic structure encodes hierarchical depth in the gene ontology

**DOI:** 10.1093/bioinformatics/btag768

**Published:** 2026-10-13

**Authors:** Sadiya Ahmad

**Affiliations:** Department of Computer Science, Iowa State University, Ames, IA 50011, United States

## Abstract

**Motivation:**

We asked whether the natural language used to name Gene Ontology (GO) Biological Process terms encodes hierarchical position in the GO directed acyclic graph.

**Results:**

Semantic representations of term names, combined with Uniform Manifold Approximation and Projection visualization and *k*-means clustering (k=20) recovered biologically coherent semantic groups whose depth distributions differed strongly (Kruskal–Wallis H=5244; $p<10^{-300}$; $\eta^{2}=.217$). Term-name length increased with depth (Pearson *r* = .34, Spearman ρ=0.37; slope =3.2 letters per level), and this positive scaling held under shortest-, longest-, and average-path depth definitions. Vocabulary entropy followed a bell-shaped diversification–specialization curve with an intermediate-depth peak (maximal at depth 4) that lies outside a Monte Carlo null envelope across most depths, and null-model tests supported a genuine linguistic–hierarchical association.

**Availability and implementation:**

The go_lingdepth Python package, regenerated result tables, and figure-generation scripts are available at https://github.com/Sadiya-ISU/Letters-and-Biological-Process and archived on Zenodo (DOI: 10.5281/zenodo.21501473).

## 1 Introduction

Biological ontologies provide controlled vocabularies for annotating genes, proteins, and phenotypes across species and experimental platforms (Ashburner *et al.* 2000, Smith *et al.* 2007). Gene Ontology (GO) is the most widely adopted such resource and organizes knowledge as a directed acyclic graph (DAG) whose terms are linked by is_a and part_of relations while also carrying short natural-language names. This dual character-graph plus curated language-suggests a concrete question: do GO term names encode information about hierarchical position?

The hypothesis is biologically plausible. Curators often inherit parent terminology when naming descendants, deeper terms require added qualifiers to distinguish increasingly specific concepts, and specialized subfields develop stable domain vocabularies. However, prior work on ontology semantics, originating in general taxonomies (Jiang and Conrath 1997, Lin 1998; Resnik 1995) and later specialized to GO (Chen *et al.* 2021, Kulmanov *et al.* 2018, Pesquita *et al.* 2009, Wang *et al.* 2007), has focused overwhelmingly on semantic similarity, graph topology, information content, and ontology-aware prediction rather than on whether the wording of term names alone reflects DAG depth. Related work in ontology engineering has supported semi-automated annotation and terminology integration (Bodenreider 2004, Jonquet *et al.* 2009), and large curated phenotype resources have grown alongside such tooling (Köhler *et al.* 2019), but not a systematic statistical analysis of linguistic structure versus hierarchical depth.

The broader idea is not unique to GO. In general lexical resources, information-content measures over the WordNet taxonomy (Miller 1995) make semantic specificity an explicit function of position in the hierarchy (Resnik 1995), and the Wikipedia category graph has been analyzed in a similar spirit (Zesch and Gurevych 2007). Yet a large biomedical ontology offers a distinct setting: terms are short, expert-curated, semantically constrained, and embedded in a formal DAG. This makes GO Biological Process an unusually strong test bed for asking whether surface language mirrors ontological structure.

This study addresses that question through a unified computational analysis of the GO Biological Process sub-ontology. It makes five contributions. First, it quantifies association between term-name length and depth. Second, it shows that sentence-embedding representations of term names recover 20 biologically coherent semantic clusters whose depth distributions differ at overwhelming significance. Third, it demonstrates that vocabulary entropy across depth follows a non-monotonic diversification–specialization pattern, rising to an intermediate-depth maximum before declining. Fourth, it validates these patterns with null models and synthetic ontologies, then proposes a minimal mechanistic hypothesis based on vocabulary inheritance, mutation, and branch specialization. Fifth, it establishes the robustness of these findings across six depth definitions, across cluster counts from k=10 to k=50, and across all three GO namespaces.

## 2 Materials and methods

### 2.1 Data acquisition and ontology depth

The go-basic.obo ontology file was downloaded from the GO (The Gene Ontology Consortium 2026) resource at https://geneontology.org. All active terms assigned to the biological_process namespace were retained, using GO:0008150 as the root.

We parsed go-basic.obo (data-version 2026–05-19; SHA-256 recorded in the Data Availability statement) with a custom parser that records, for every non-obsolete term, its identifier, name, namespace, is_a parents, and part_of parents as separate relation sets. Terms flagged “is_obsolete: true” were excluded in full: they contribute no name, namespace, or edge, and are removed again when namespace membership is resolved. Only the single primary name: field of each term entered the linguistic analysis; the synonym: and alt_id: fields are never read by the parser. Each term therefore contributes exactly one label, and vocabulary diversity cannot be inflated by alternative wordings of the same concept. Ontology depth was computed three ways from the namespace root (GO:0008150 for Biological Process): the shortest path, the longest path, and the average length over all distinct root-to-term paths, each evaluated over both the is_a and is_a   part_of edge sets. The shortest-path definition over is_a   part_of is used for the primary figures; the longest- and average-path results are reported as a robustness check (§3.1, Supplementary §S1, Table S1, available as supplementary data at *Bioinformatics* online), because terms with multiple parents can otherwise reach the root through curated shortcut links. Under the primary definition, the root has depth 0, direct children depth 1, and Biological Process terms span depths 0–13 (24,136 non-obsolete terms in this release).

### 2.2 Linguistic representations

Each GO term name was treated as a short text sequence. For the vocabulary-entropy analysis (Equation 3), we tokenize each name into lowercase unigrams [regex (a-z0-9) , tokens of length >1]. Because GO names contain ubiquitous generic tokens such as “process,” “regulation,” and “cell,” we report entropy under three stop-word policies-none, the scikit-learn English list, and English plus a curated GO-generic list-and verify that the depth profile and the depth-4 peak are unchanged across all three (§S4, Fig. S4, available as supplementary data at *Bioinformatics* online). The curated GO-generic list is (activity, biological, cell, cellular, dependent, function, involved, mediated, molecular, negative, positive, process, regulated, regulating, regulation, response, system, via).

Dense semantic sentence embeddings were generated for each GO term name using the sentence-transformer model all-MiniLM-L6-v2 (Reimers and Gurevych 2019). These embeddings provide a compact contextual representation of each term name and were used for clustering and visualization.

### 2.3 Dimensionality reduction and clustering

Semantic clustering was performed on the transformer embeddings. The dense sentence embeddings were first reduced to 50 dimensions using principal component analysis (PCA), producing a compact representation suitable for clustering while preserving the dominant structure of the embedding space.

Unsupervised grouping was then performed with *k*-means using k=20 clusters on the PCA-reduced embeddings. The cluster count *k* was examined over the range 10–50 using the silhouette score (Rousseeuw 1987), the Davies–Bouldin index (Davies and Bouldin 1979), and the elbow (inertia) criterion (§S2, Fig. S2, available as supplementary data at *Bioinformatics* online). These internal validity indices do not identify a decisive optimum in this range and, if anything, mildly favor larger *k*; we therefore do not present k=20 as a metric-selected optimum. We retain k=20 because it yields interpretable, non-redundant biological themes (Table 1), and §S2, available as supplementary data at *Bioinformatics* online (Table S2, available as supplementary data at *Bioinformatics* online) shows that the depth-by-cluster effect remains highly significant at every *k* in this range ($\eta^{2}=.168$–.252). Cluster labels were assigned *post hoc* by a single annotator from a fixed, seeded random sample of 10 term names drawn from each cluster, the label recording the biological theme shared by that sample and the vocabulary recurring across it. The labels serve only to make (Table 1) readable: no reported statistic depends on them-every value, including the depth-by-cluster Kruskal–Wallis test and its effect size, is computed from numeric cluster identity and is unchanged if the labels are permuted or removed entirely. Because their role is confined in this way, this interpretive step was not put through independent validation: no second annotator, held-out sample, or external reference vocabulary was used.

**Table 1:** Biological themes inferred from *k*-means clustering (k=20) of sentence-transformer embeddings.^a^

Cluster	Inferred biological theme	Cluster	Inferred biological theme
0	Organelle assembly and macromolecular complex organization	10	Immune cell activation and antigen processing
1	Nervous system development and axon guidance	11	Cell migration, adhesion, and differentiation
2	Protein localization and membrane dynamics	12	Small-molecule and secondary metabolism
3	Transcription, RNA processing, and DNA replication	13	Cell cycle progression and immune regulation
4	Regulatory processes (metabolic and developmental)	14	Vesicle trafficking and membrane transport
5	Hormone signaling and xenobiotic response	15	Organ morphogenesis and tissue patterning
6	Biosynthetic and anabolic metabolic processes	16	Transmembrane ion transport and secretion
7	Muscle contraction and cardiovascular physiology	17	Catabolic and degradative metabolic pathways
8	Mitosis, meiosis, and chromosome segregation	18	Intracellular signal transduction cascades
9	Developmental morphogenesis and pattern formation	19	Synaptic transmission and neurotransmission

a Labels were assigned post hoc by inspecting a seeded random sample of 10 term names per cluster (Section 2.3); they are descriptive only and no reported statistic depends on them.

For visualization, Uniform Manifold Approximation and Projection (UMAP; Becht *et al.* 2018, McInnes *et al.* 2018) was applied to the same PCA-reduced embedding space using n_neighbors=15, min_dist=0.1, and a fixed random seed. Importantly, clustering was performed in the higher-dimensional PCA space, not on the two-dimensional UMAP coordinates, so the visual projection serves only as an interpretable display of the semantic organization.

### 2.4 Statistical analyses

Depth distributions across clusters were compared by the Kruskal–Wallis test (Kruskal and Wallis 1952)


(1) $$H=\frac{12}{N(N\;1)}\sum_{i=1}^{k}n_{i}{\left({\bar{R}}_{i}-\frac{N\;1}{2}\right)}^{2}$$

where $n_{i}$ and ${\bar{R}}_{i}$ denote the size and mean rank of cluster *i* and $N=\sum_{i}n_{i}$. Length–depth association was measured with Pearson *r* and Spearman ρ, and an ordinary least-squares fit of the form


(2) $$\hat{\text{letters}}=\beta_{1}\cdot \text{depth}\;\beta_{0}$$

was used to summarize scaling.

Vocabulary diversity at depth *d* was measured with Shannon (1948) entropy


(3) $$H(d)=-\sum_{i}p_{i}(d){\;\text{log}\;}_{2}p_{i}(d)$$

where $p_{i}(d)$ is the empirical frequency of token *i* among all tokens appearing in terms at depth *d*. The depth-wise entropy profile is summarized descriptively. We report the empirical peak depth with a leave-one-depth-out stability interval and assess significance with a Monte Carlo null envelope (§2.5, item 3; §3.4, available as supplementary data at *Bioinformatics* online) rather than by parametric model selection, since only 14 depth levels are available. A linear/logarithmic/quadratic overlay is retained for illustration only and is shown, with distinct line styles, in Fig. S5, available as supplementary data at *Bioinformatics* online.

### 2.5 Validation design

To test whether the observed linguistic–hierarchical relationships could arise by chance, we used two null-model perturbations, one entropy-specific Monte Carlo resampling procedure, and a family of synthetic ontology benchmarks.

1. *Random label permutation*: term names were randomly reassigned to fixed ontology nodes while preserving the original DAG structure and the overall corpus of names. This destroys the true mapping between language and hierarchy while keeping both the graph and the set of names unchanged.
2. *Random depth reassignment*: integer depth labels were randomly permuted across terms while leaving names at their original nodes. This preserves the observed vocabulary at each node but breaks the association between that vocabulary and true hierarchical position.
3. *Monte Carlo entropy resampling*: for entropy, repeated random term-to-node assignments were generated and the depth-wise entropy curve was recomputed after each resampling. This produced a null distribution of entropy at each depth and therefore a depth-specific null envelope against which the empirical entropy profile could be compared.
4. *Synthetic ontologies*: to assess sensitivity and specificity under controlled conditions, we generated simplified GO-like hierarchical graphs with multiple depth levels and branching structure resembling the broad organization of Biological Process. Simulated term names were then assigned under three generative regimes. In the *signal* regime, child nodes inherited part of the vocabulary of their parent branch and gained additional branch-specific specialized tokens with increasing depth. In the *noise* regime, names were generated independently of depth and branch. In the *mixed* regime, part of the vocabulary followed the signal process and part was assigned randomly. The full downstream analysis pipeline was then rerun on these synthetic structures to determine whether the observed length–depth and entropy–depth patterns were recoverable only when hierarchical linguistic structure was truly present.

Together, these validation procedures distinguish genuine coupling between ontology language and hierarchical depth from artifacts caused by corpus composition, random assignment, or generic graph shape.

### 2.6 Algorithm

#### 2.6.1 Analysis pipeline

The computational workflow can be summarized as follows:

1. Parse go-basic.obo and retain all Biological Process terms.
2. Build the DAG induced by is_a and part_of relations and compute shortest-path depth from GO:0008150 (Section 2.1 gives the longest- and average-path variants).
3. Generate dense semantic sentence embeddings of term names using all-MiniLM-L6-v2.
4. Reduce the dense embedding matrix to 50 dimensions using PCA.
5. Cluster the reduced embeddings with *k*-means (k=20).
6. Visualize the same reduced semantic space with UMAP in two dimensions.
7. Quantify the relation between ontology depth and language using letter-count regression, per-cluster depth comparisons, and depth-wise Shannon entropy.
8. Validate the observed structure using permutation-style null models and synthetic ontology experiments.

This separation is important. Ontology depth is not used to construct the embedding space or to assign clusters; it is introduced only afterward for evaluation. Consequently, any association between cluster identity and depth indicates that GO term names alone carry recoverable hierarchical information.

### 2.6.2 Vocabulary-evolution model

To interpret the observed entropy profile, we formulate a minimal vocabulary-evolution model with three mechanisms. We stress at the outset that this is a mechanistic hypothesis offered as a candidate explanation, not a generative model fitted to the GO. Its parameters are not estimated from GO, it is not evaluated against the ontology’s documented revision history, and none of the empirical results in Section 3 depend on it.

1. *Vocabulary inheritance*: a child node reuses a subset of parent tokens, so that
  $$V_{\text{child}}⊇S\subseteq V_{\text{parent}}.$$
2. *Vocabulary mutation*: a child introduces new tokens with probability $p_{m}$ per token position.
3. *Branch specialization*: different top-level branches maintain partially distinct lexicons, reducing cross-branch token sharing.

If I(d) denotes the vocabulary-introduction rate at depth *d* and R(d) denotes the reuse rate, entropy evolves approximately as


(4) H(d)≈I(d)−R(d)

When branching is rapid, I(d) rises and diversity increases; once branches mature, reuse dominates and diversity declines.

Under these assumptions the resulting depth profile would take the illustrative form.


(5) $$H(d)∼a\;d-b\;d^{2},\;a,b>0$$

with an entropy peak at intermediate depth. Here a,b>0: the linear term a·d drives the initial rise in lexical diversity as branches proliferate, while the quadratic penalty $-b\cdot d^{2}$ produces the eventual decline as mature branches reuse a narrowing vocabulary. Together they place a single interior maximum at $d^{*}=a/2b$ (Equation 6)—an intermediate-depth entropy peak rather than monotonic growth.


(6) $$d^{*}=\frac{a}{2b}$$

## 2.7 Implementation

All analyses were implemented in Python. Ontology parsing and depth computation were performed with a custom workflow for extracting Biological Process terms and their is_a and part_of relations from go-basic.obo. The depth-wise vocabulary entropy (Equation 3) is computed from lowercase unigram tokens under the three stop-word policies defined in Section 2.2 (§S4, Table S4, Fig. S4, available as supplementary data at *Bioinformatics* online).

Dense semantic representations were generated with the sentence-transformer model sentence-transformers/all-MiniLM-L6-v2. These embeddings were reduced with PCA to 50 dimensions and clustered with *k*-means (k=20) using scikit-learn (Pedregosa *et al.* 2011), then visualized with UMAP. The implementation therefore separates token-level vocabulary analysis from transformer-based semantic clustering while keeping both components tied to the same curated set of GO term names.

This design keeps the workflow lightweight and reproducible. It does not require graph neural networks or ontology-specific embedding architectures for the main result; instead, it tests how much hierarchical signal is recoverable from the language of term names alone.

## 3 Results

### 3.1 Term-name length scales with depth

Term-name letter count increases with ontology depth (Fig. 1). The observed correlation is moderate but highly robust (Pearson r=.339, Spearman ρ=0.366, both p≪.001), and the fitted regression.


(7) $$\hat{\text{letters}}\approx 3.22\times \text{depth}\;19.68$$

indicates that each additional level adds about three letters on average. The spread of points and the widening depth-wise distributions show substantial within-level variance, but the central trend remains clear: deeper concepts require longer and more qualified linguistic descriptions. This positive length–depth association is robust to how depth is defined. Recomputing over the is_a   part_of edge set under the longest-path and average-path metrics—the latter averaging over all root-to-term paths and thus down-weighting shallow shortcut edges—yields Spearman correlations of ρ=0.276 and ρ=0.344, respectively, compared with ρ=0.366 for the shortest path; across all six edge-set × metric combinations, ρ stays within 0.27–0.40 (Table S1, Fig. S1, available as supplementary data at *Bioinformatics* online). The fitted slope is more metric-sensitive, ranging from 1.68 to 3.25 letters per level; the lowest value arises under the longest-path definition, which extends the depth axis from 13 to 17 levels without lengthening the names. The intermediate-depth entropy peak is likewise present under all six definitions, at depth 4 for four of them and at depths 6 and 9 under the average- and longest-path variants of “is_a   part_of.” The sign, significance, and interior-peak structure of the relationship are, therefore, robust to how depth is defined, while its steepness and the exact peak location are not.

**Figure 1:**
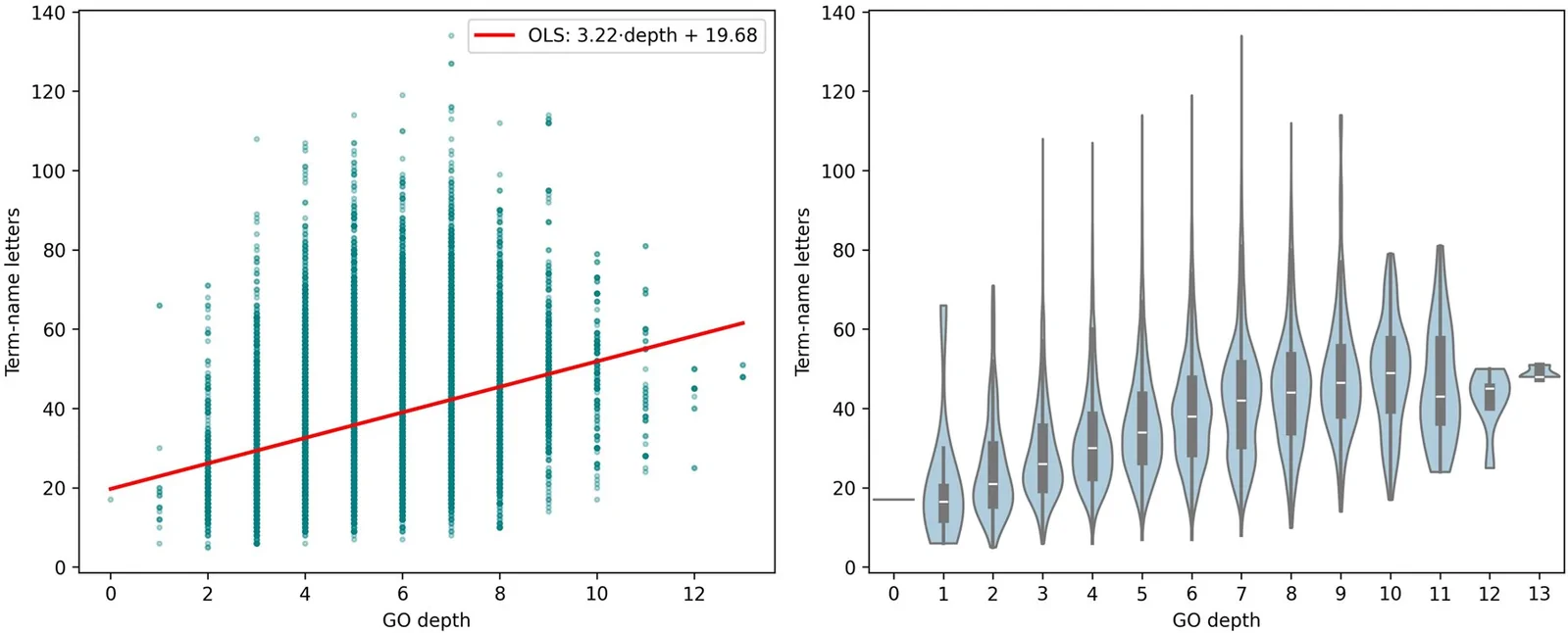
Length–depth relationship in GO Biological Process. The scatter plot shows positive global association, while the violin plots show an upward shift and wider spread in term-name letter count at greater depths.

### 3.2 Semantic clustering recovers biologically coherent subdomains

Unsupervised clustering of GO Biological Process term names partitions the ontology into recognizable functional groupings spanning development, metabolism, immunity, signaling, cell-cycle control, membrane transport, and neurophysiology. Because the clustering is driven by linguistic representations of the term names rather than by explicit graph topology, this result shows that short curated names alone contain substantial biological and structural information.

The low-dimensional projection (Fig. 2, left) further indicates that the semantic landscape is not purely gradual. Distinct two-dimensional groupings are visible, and these align closely with the cluster identities assigned in the higher-dimensional embedding space. The cluster-depth boxplots (Fig. 2, right) show that linguistically defined groups occupy significantly different regions of the ontology, although the depth distributions overlap substantially. Thus, semantic grouping is associated with hierarchical position, but not in the form of a strict shallow-versus-deep separation for all biological themes.

**Figure 2:**
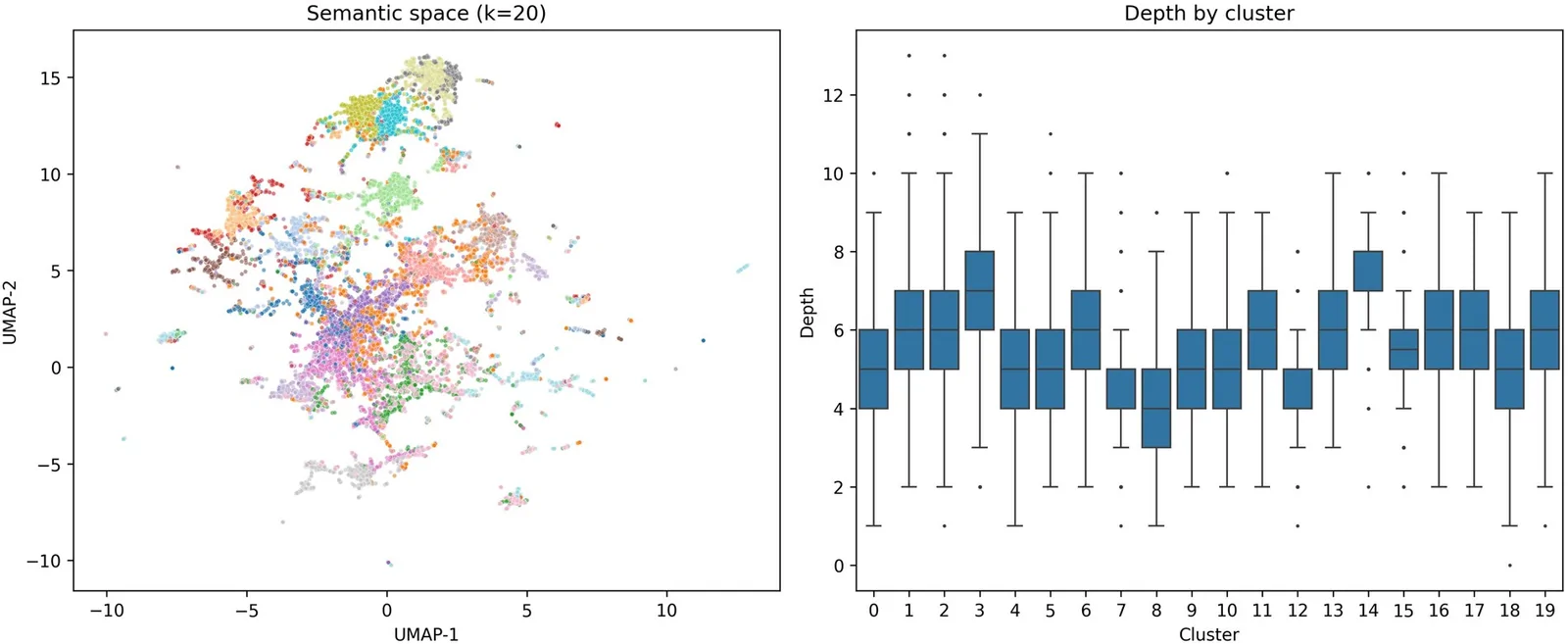
Semantic organization of GO Biological Process language. Left: UMAP projection of PCA-reduced sentence-transformer embeddings of GO term names, showing separated semantic groupings in linguistic space. Right: boxplots of ontology depth by cluster, indicating that linguistically defined groups occupy significantly different, though overlapping, depth ranges in the Biological Process DAG.

The difference across clusters is statistically strong:


$$H=5244,\;\text{df}=19,\;p<10^{-300}\;(\eta^{2}=.217)$$

This result demonstrates linguistic–structural coupling: term-name language alone is sufficient to recover semantic groups whose depth distributions differ systematically across the GO Biological Process DAG. The effect size $\left(\eta^{2}=.217\right)$ indicates moderate practical significance of the cluster-depth association despite the large test statistic due to sample size.

### 3.3 Entropy follows a diversification–specialization profile

Vocabulary entropy across depth shows a bell-shaped descriptive pattern rather than a simple monotonic trend. At levels 0–1, entropy is low because only a few very broad terms exist. At Levels 2–4, rapid branching introduces multiple domain-specific vocabularies simultaneously and entropy rises. At Levels 5–13, entropy declines as terms become increasingly specialized within established domains and reuse a narrower set of characteristic tokens.

The entropy–depth Spearman correlation is weak and nonsignificant (ρ=−0.29, p=.32), so the data do not support a simple monotonic association—consistent with, but not in itself establishing, a nonmonotonic profile. The substantive evidence for a genuine rise-then-fall pattern is the directly observed peak and a Monte Carlo null envelope, not a parametric fit. Empirically, vocabulary entropy rises to a maximum at depth 4 (9.22 bits under the English stop-word policy; the same peak depth under all three stop-word policies, §S4, available as supplementary data at *Bioinformatics* online) and declines thereafter. Against the depth-wise Monte Carlo null obtained by repeated random term-to-node reassignment (§3.4, Fig. 3, right), the observed entropy falls outside the 1–99% null band at depths 1 and 3–13 (12 of the 14 depths). This is distribution-free evidence that requires no parametric form. We therefore treat the quadratic curve in Fig. 3 purely as a descriptive guide (its vertex falls at depth ≈6.24, stable to a leave-one-depth-out range of 5.7–6.4, consistent with an interior peak) and do not claim it is the statistically preferred model—with only ∼14 depth levels, such a comparison would be unreliable.

**Figure 3:**
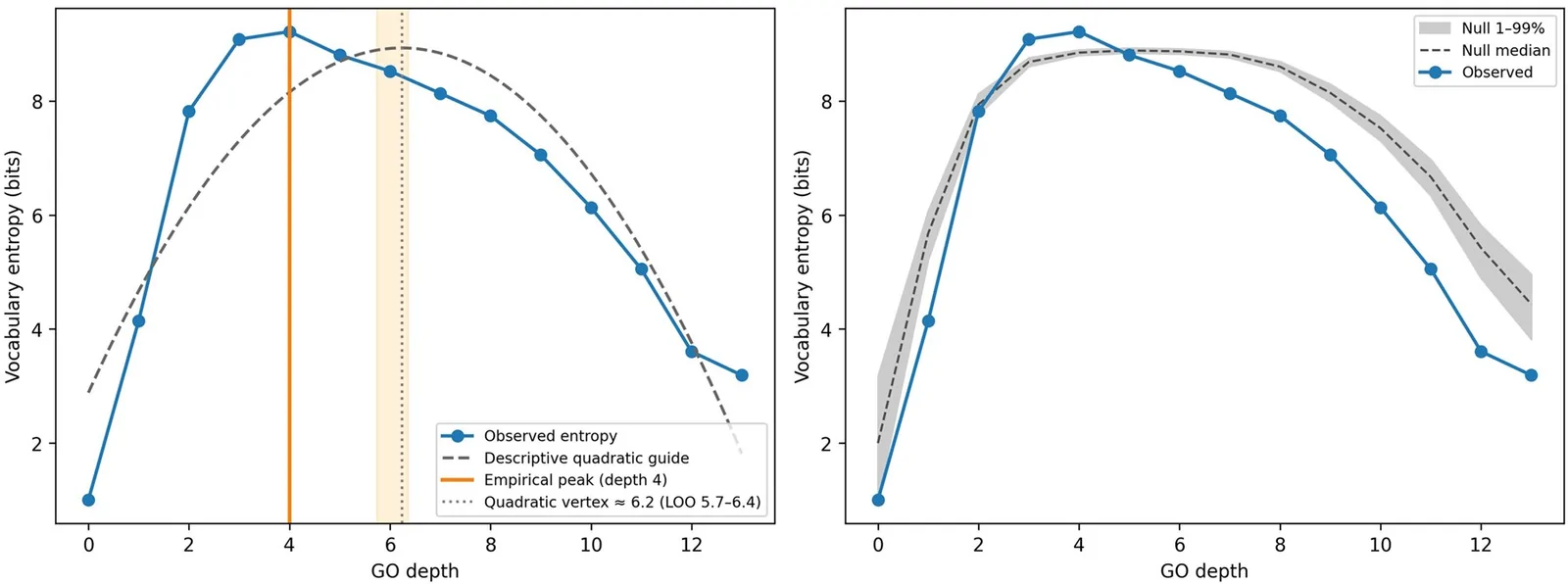
Entropy analysis across the ontology hierarchy. Left: empirical vocabulary entropy by GO depth, showing a bell-shaped diversification–specialization profile with maximal diversity at intermediate levels. Right: depth-wise Monte Carlo null envelopes obtained by repeated random term-to-node reassignment, showing that the observed entropy curve is not reproduced under random mappings between ontology structure and term names.

### 3.4 Null models and synthetic ontologies support a genuine effect

The observed linguistic–hierarchical relationships depend on preserving the correct mapping between ontology structure and term names. Random label permutation destroys the length–depth association and disrupts the empirical entropy profile, showing that corpus statistics alone are insufficient. Random reassignment of depth labels likewise abolishes the signal, demonstrating that the effect depends on the actual hierarchy rather than on local word-frequency structure alone.

For entropy, the null analysis was performed by repeated random term-to-node reassignment, with the depth-wise entropy curve recomputed after each resampling. This procedure yields a null distribution for entropy at each depth and therefore a depth-specific null envelope. Under this resampling scheme, the observed entropy profile falls outside the corresponding null range across the analyzed depths, indicating that the diversification–specialization pattern is unlikely to be a sampling artifact.

Sensitivity and specificity were further examined using synthetic GO-like ontologies (Fig. 4, left). Noise-only ontologies yield near-zero length–depth correlation (mean =0.00). Mixed ontologies produce intermediate correlations (mean =0.46). Signal ontologies yield strong correlations (mean =0.90). These distributions are well separated, indicating that the framework can detect genuine hierarchical linguistic structure while avoiding false positives when no such structure is present. Under the vocabulary-evolution model of §2.6.2, simulated term-name length likewise grows with simulated depth (Fig. 4, right). This demonstrates only that the hypothesized mechanisms are sufficient to produce the observed direction of scaling; it does not establish that they are the mechanisms that generated GO, and no alternative generative process was fitted and compared.

**Figure 4:**
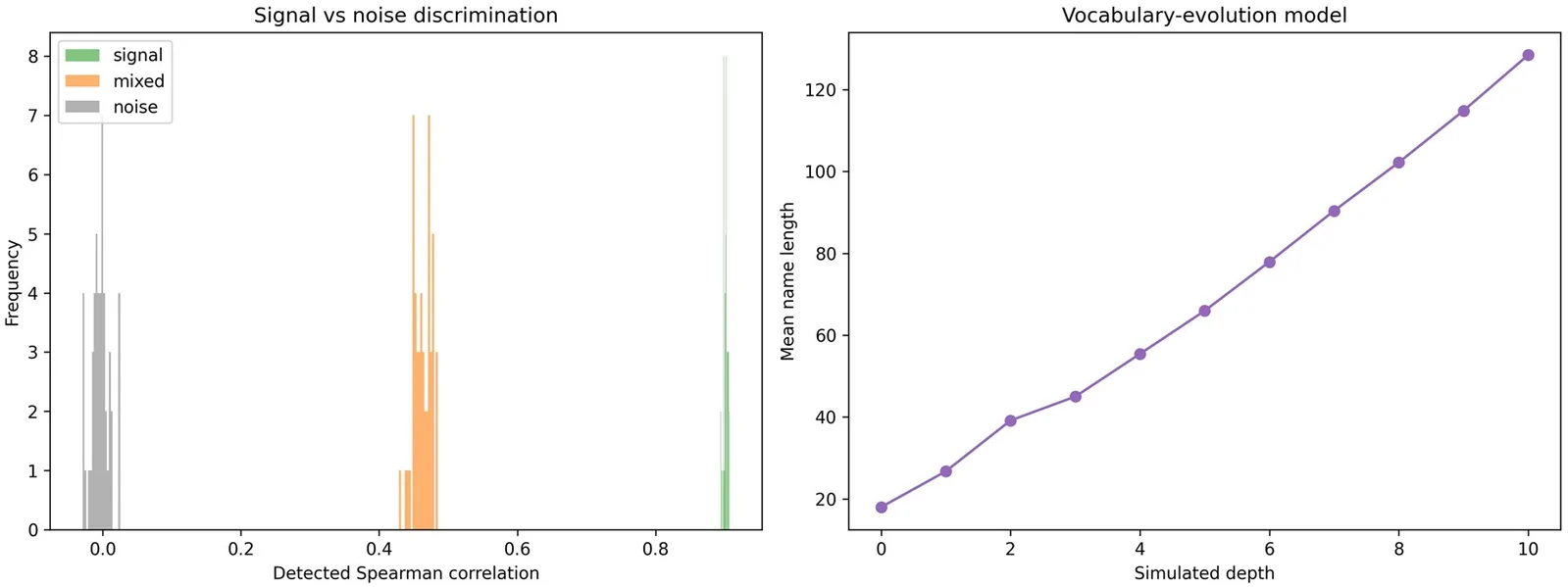
Validation and mechanistic explanation. Left: distributions of detected length–depth correlations in synthetic GO-like ontologies under signal, mixed, and noise regimes, showing clear separation between true-signal and null settings. Right: mean simulated term-name length rising with depth under the proposed vocabulary-evolution model, in which token inheritance, mutation, and branch-specific specialization can reproduce the growth of name length with ontology depth.

## 4 Discussion

### 4.1 A diversification–specialization characteristic of ontology language

The combined empirical and synthetic evidence motivates a general-but topology-modulated principle: GO vocabulary evolves from a low-diversity root lexicon, through an intermediate phase of maximal branch diversification, to deep specialization in which branch-specific vocabularies are repeatedly reused. We tested this directly by rerunning the full pipeline on the other two namespaces (§S3, Fig. S3, Table S3, available as supplementary data at *Bioinformatics* online). The intermediate-depth entropy peak appears in all three namespaces—at Depth 4 in Biological Process, Depth 5 in Molecular Function, and Depth 2 in Cellular Component-and the depth-by-cluster effect is significant in each (Kruskal–Wallis H=5244/1576/319; k=20; $\eta^{2}=.217/.155/.074$). Its position and intensity, however, are clearly modulated by each namespace’s branching topology: Molecular Function gives a clean interior arc, whereas Cellular Component-a shallower, more compressed hierarchy with much weaker length scaling (ρ=0.100)-rises by depth 2, plateaus through depth 4, and declines thereafter, an earlier and more compressed version of the same shape.

These differences track systematic contrasts in how the three namespaces are built and named. Biological Process names are strongly compositional: a child term’s name typically embeds its parent’s name and adds a qualifier, as in “positive regulation of X” beneath “regulation of X.” Descending one level mechanically appends letters while reusing the parent’s tokens-precisely the behavior that produces strong length scaling (ρ = 0.366) alongside deep-level vocabulary reuse. Cellular Component names are instead largely self-contained: a child usually names a physically distinct component rather than a modified version of its parent (“nucleolus” is part of “nucleus”), and names are correspondingly short noun phrases. This reflects a difference in the underlying relation: Cellular Component is largely a partonomy. Because component names do not accumulate inherited qualifiers, depth is weakly encoded in name length (ρ = 0.100). Molecular Function is intermediate, with the frequent use of the suffix “activity” that limits both length growth and vocabulary turnover (ρ=0.176). Cellular Component is also by far the smallest and most compressed of the three (4075 terms across Depths 0–10, against 24 136 across 0–13), so fewer terms per level and a narrower depth range further attenuate both the length correlation and the cluster–depth effect size.

The defensible thesis is, therefore, that the rise-then-fall entropy signature is a general feature of expert-curated GO vocabulary, while where along the hierarchy it peaks and how sharply is a topology-dependent property of each namespace rather than a fixed universal law.

### 4.2 Limitations, implications, and future directions

Depth in a multiparent DAG admits several definitions; rather than rely on a single choice, we computed shortest-, longest-, and average-path depth over both the is_a and is_a   part_of edge sets, and confirmed that the length–depth scaling and the intermediate-depth entropy peak hold under all of them, with the peak location varying between Depths 4 and 9 (§3.1, Supplementary §S1, available as supplementary data at *Bioinformatics* online). The analysis spans all three GO namespaces (§4.1, Supplementary §S3, available as supplementary data at *Bioinformatics* online); the entropy signature generalizes beyond Biological Process, though its peak position and intensity are topology dependent, so we characterize it as a general-but-modulated property rather than a strict universal law. A remaining limitation is that unigram entropy is insensitive to word order and to contextual semantics beyond sparse token co-occurrence. A second is the status of the vocabulary-evolution model of §2.6.2: it is a conceptual account of how inheritance, mutation, and branch specialization could produce a rise-then-fall entropy profile, not a formally validated evolutionary model of GO. Its parameters were not fitted to ontology data, it was not tested against alternative generative processes, and it makes no calibrated quantitative predictions. Establishing it as a generative model would require fitting it to successive GO releases and testing its predictions on held-out ontology growth—work we identify as a natural next step rather than a claim of this paper.

Even with those limitations, the practical implications are substantial. Because graph access is not required to obtain a meaningful signal, term-name language could support automated depth prediction, vocabulary-based quality auditing, and depth-aware semantic representations that complement graph-based similarity. Future work should compare other biomedical ontologies and evaluate contextual embeddings such as BioBERT (Lee *et al.* 2020) and PubMedBERT (Gu *et al.* 2022). Longitudinal analysis of GO additions may also reveal whether the diversification–specialization peak shifts as the ontology matures.

In summary, GO Biological Process is both a graph of biological concepts and a structured linguistic artifact: its term names encode hierarchical position, semantic domain membership, and biological specificity in a measurable and reproducible way.

## Supplementary Material

btag768_Supplementary_Data

## Data Availability

The GO go-basic.obo ontology analyzed in this study is openly available from the Gene Ontology Consortium at [https://geneontology.org], the exact pinned release used here—data-version releases/2026–05-19, SHA-256 923645000afcb1df0e72e252b64950b1c2bcc98c9393fec8c1dd1a0f7c9d4d77—is archived at https://release.geneontology.org/2026-05-19/. All analysis code, the regenerated result tables, and the figure-generation scripts are released as the tested, pip-installable Python package go_lingdepth at https://github.com/Sadiya-ISU/Letters-and-Biological-Process and archived on Zenodo (DOI: 10.5281/zenodo.21501473), §S6, available as supplementary data at *Bioinformatics* online, records the full computational environment and the determinism settings.
