# Supplementary material for: Letters and biological process: linguistic structure encodes hierarchical depth in the gene ontology
Source: Bioinformatics. 2026 Oct 13;42(10):btag768. doi: 10.1093/bioinformatics/btag768 (PMC13718435; doi:10.1093/bioinformatics/btag768)
Supplement: Supplementary file 1 [file btag768_supplementary_data.pdf]

Supplementary Material

Letters and Biological Process: Linguistic Structure Encodes Hierarchical Depth in the Gene Ontology

All values were regenerated from the Gene Ontology release **go-basic, releases/2026-05-19** (SHA-256 923645000afcb1df0e72e252b64950b1c2bcc98c9393fec8c1dd1a0f7c9d4d77).

Random seeds are fixed throughout; set `PYTHONHASHSEED=0` for exact bit-reproduction.

Source tables: `results/depth_robustness.csv`, `results/kselection.csv`, `results/namespace_summary.csv`, `results/namespace_entropy_by_depth.csv`, `results/entropy_by_depth.csv`, `results/entropy_null_envelope.csv`, `results/entropy_model_summary.json`, `results/null_corr_summary.json`, `results/synthetic_corrs.csv`.

S1. Robustness to the ontology-depth definition

In a directed acyclic graph with multiple parentage, "depth" is not unique. We computed depth three ways from the Biological Process root (GO:0008150) — the **shortest** path, the **longest** path, and the **average** length over all distinct root-to-term paths — each over both the `is_a` and the `is_a+part_of` edge sets. The average-path metric is of particular interest because, by averaging over every root-to-term path, it actively down-weights the shallow "shortcut" edges that a shortest-path definition could exploit.

Table S1. Length–depth association and entropy-peak depth across depth definitions (Biological Process; n = 24,136 terms). Pearson r and Spearman ρ are between term-name character count and depth; the OLS slope is in letters per depth level; the entropy peak is the depth at which depth-wise vocabulary entropy is maximal.

Edge set	Metric	Max depth	Pearson r	Spearman ρ	OLS slope	Entropy-peak depth
**`is_a+part_of`**	shortest (**primary**)	13	0.339	**0.366**	3.22	**4**
**`is_a+part_of`**	longest	17	0.268	0.276	1.68	9
**`is_a+part_of`**	average	13.8	0.334	0.344	2.75	6
**`is_a` only**	shortest	13	0.347	0.371	3.25	4
**`is_a` only**	longest	16	0.359	0.376	2.37	4
**`is_a` only**	average	13.8	0.377	0.395	2.99	4

All Pearson and Spearman p-values are $< 10^{-300}$ (reported as p = 0 by the solver).

Narrative. The length–depth association is **positive under every definition**, with Spearman ρ confined to **0.27–0.40** across all six edge-set × metric combinations. The vocabulary-entropy profile retains an **intermediate-depth peak** in every case (peak depth 4–9; depth 4 for four of the six definitions). The reported relationships are therefore not artifacts of the shortest-path choice. **Fig. S1** visualizes the length–depth Spearman ρ and the entropy-peak depth across the three metrics for both edge sets.

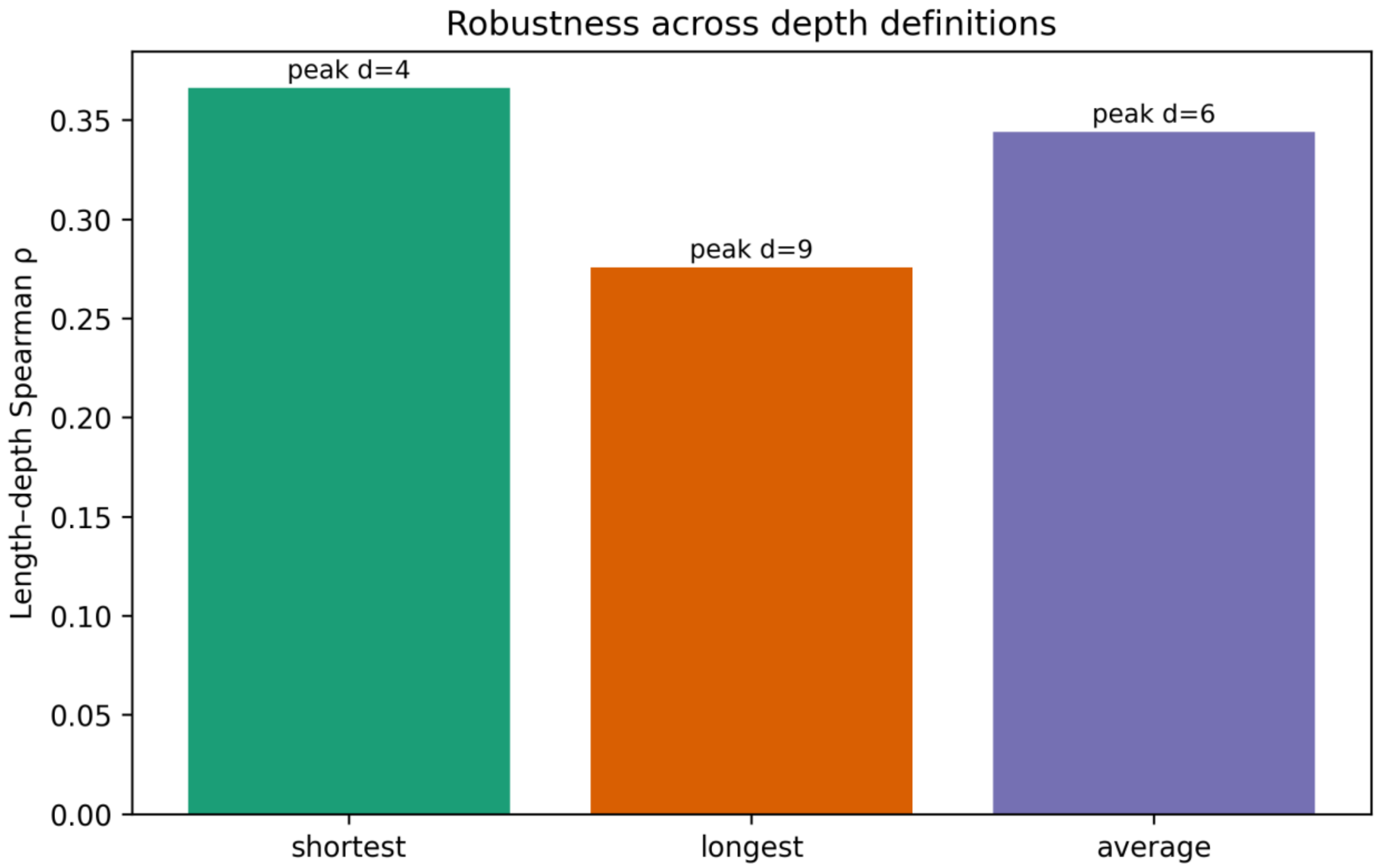

Figure S1. Length–depth Spearman ρ and entropy-peak depth across the shortest, longest, and average path-depth metrics for both edge sets.

Alt text: Bar chart of length-depth Spearman correlation under shortest, longest, and average path depth definitions, all positive and similar, each annotated with its intermediate entropy-peak depth.

S2. Choice of the cluster number k

We examined k over 10–50 using internal cluster-validity indices (silhouette score, Davies–Bouldin index, k-means inertia/elbow) and the substantive depth-by-cluster Kruskal–Wallis test.

Table S2. Cluster-validity indices and the depth-by-cluster Kruskal–Wallis test versus k (Biological Process).

k	Inertia	Silhouette	Davies–Bouldin	KW H	KW p	η^2
**10**	9245.2	0.074	2.979	4067	$< 10^{-300}$	0.168
**15**	8597.0	0.079	2.739	4313	$< 10^{-300}$	0.178
**20**	**8224.9**	**0.076**	**2.799**	**5244**	**$< 10^{-300}$**	**0.217**
**25**	7948.0	0.079	2.690	5087	$< 10^{-300}$	0.210
**30**	7706.3	0.080	2.664	5584	$< 10^{-300}$	0.230
**35**	7492.1	0.085	2.654	5356	$< 10^{-300}$	0.221
**40**	7323.8	0.085	2.631	5534	$< 10^{-300}$	0.228
**45**	**7153.4**	**0.086**	**2.588**	**5819**	**$< 10^{-300}$**	**0.240**
**50**	7036.3	0.084	2.607	6126	$< 10^{-300}$	0.252

Narrative. The internal validity indices **do not identify a decisive optimum** in this range: silhouette is low and nearly flat (≈ 0.07–0.085, best near k = 45), Davies–Bouldin improves and is best near k = 45, and inertia shows no sharp elbow. If anything they **mildly favour larger k**, so we do **not** present k = 20 as a metric-selected

optimum. What is decisive is robustness: the depth-by-cluster Kruskal–Wallis test is significant at **$p < 10^{-300}$ at every k**, with effect size **η^2 between 0.17 and 0.25**. We retain k = 20 (H = 5244, η^2 = 0.217) because it yields interpretable, non-redundant biological themes (main-text Table 1) while sitting squarely within this stable region; the central finding does not depend on the specific value. **Fig. S2** plots the four indices against k.

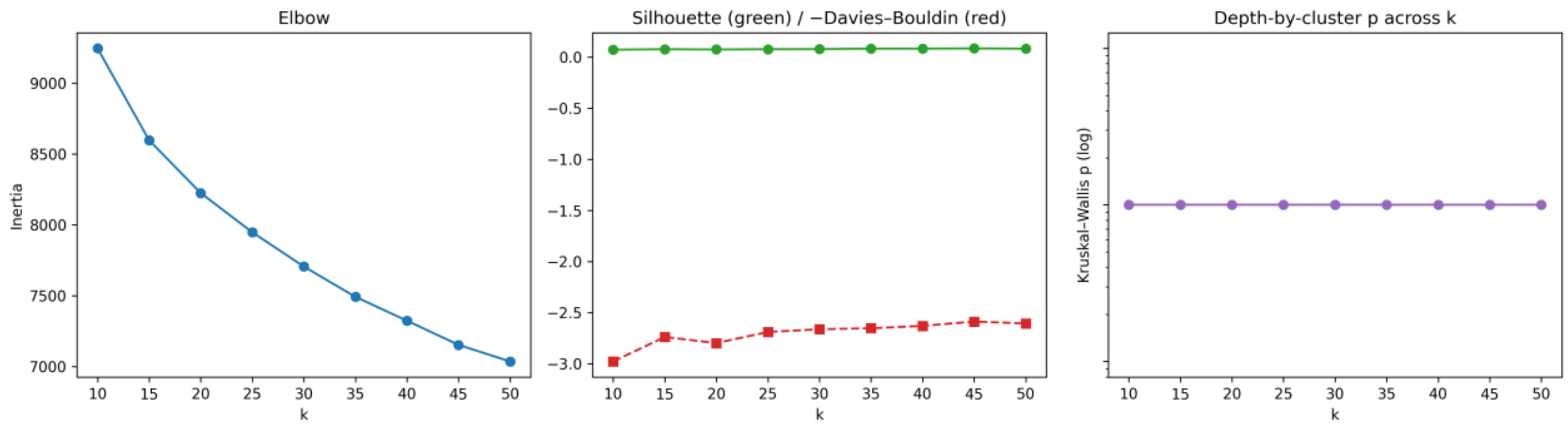

Figure S2. Cluster-validity indices (inertia, silhouette, Davies–Bouldin) and the depth-by-cluster Kruskal–Wallis significance versus k (10–50).

Alt text: Three panels: k-means inertia versus k (elbow); silhouette and negative Davies-Bouldin versus k; and Kruskal-Wallis significance versus k, remaining astronomically small for every k from 10 to 50.

S3. Generalization to Molecular Function and Cellular Component

We reran the full pipeline (depth, length, entropy, PCA-embedding clustering) on the Molecular Function and Cellular Component namespaces.

Table S3. Cross-namespace summary. Length ρ is the Spearman correlation between term-name length and depth (shortest-path, `is_a+part_of`); the entropy-peak depth is where depth-wise vocabulary entropy is maximal; KW H and η^2 are the depth-by-cluster Kruskal–Wallis statistic and effect size at k = 20.

Namespace	n terms	Max depth	Length ρ	OLS slope	Entropy-peak depth	KW H (k = 20)	η^2
**Biological Process**	24,136	13	0.366	3.22	4	5244	0.217
**Molecular Function**	10,052	9	0.176	1.87	5	1576	0.155
**Cellular Component**	4,075	10	0.100	0.63	2	319	0.074

(For Biological Process the depth-by-cluster statistic is the canonical k-selection run, H = 5244 / η^2 = 0.217; see §S2.)

Narrative. The intermediate-depth entropy peak appears in **all three namespaces** (Biological Process depth 4, Molecular Function depth 5, Cellular Component depth 2), so the rise-then-fall signature is **general**, not a Biological-Process idiosyncrasy. Its **position and intensity are topology-modulated**, however. Molecular Function gives a **clean interior arc** (entropy rising to a single maximum at depth 5 and declining smoothly). Cellular Component is a shallower, more compressed hierarchy with much **weaker length scaling (ρ = 0.100)**: entropy rises sharply by depth 2, plateaus through depth 4, then declines through depth 10 — an **earlier and more compressed** version of the same shape. The defensible claim is therefore a **general-but-topology-**

modulated diversification–specialization property rather than strict universality. **Fig. S3** overlays the depth-wise entropy curves for the three namespaces.

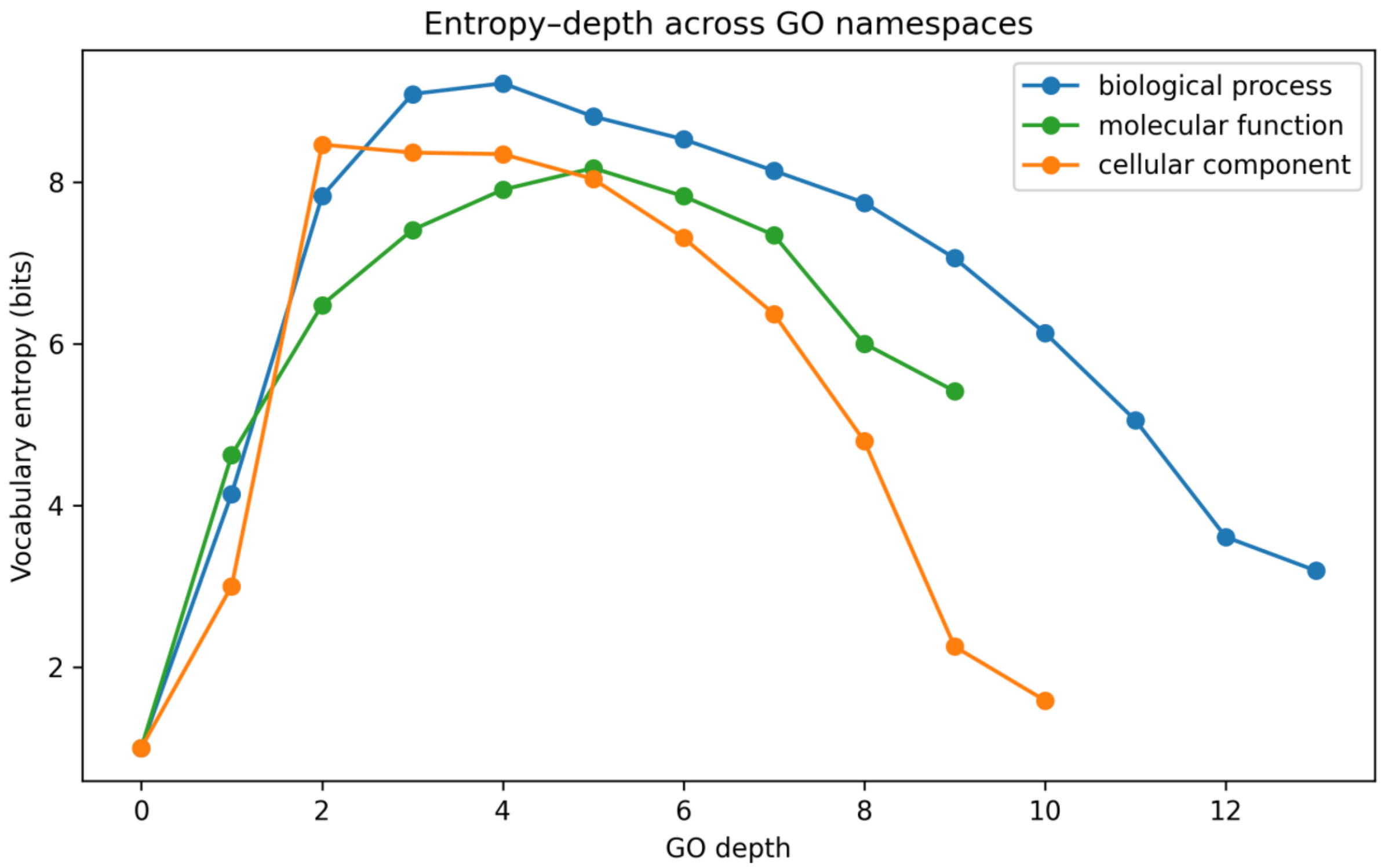

Figure S3. Depth-wise vocabulary entropy for the Biological Process, Molecular Function, and Cellular Component namespaces.

Alt text: Vocabulary-entropy curves over depth for the three GO namespaces, each showing an intermediate-depth diversification peak at a topology-dependent position.

S4. Stop-word sensitivity of the entropy profile

Depth-wise vocabulary entropy (Eq. 3) is computed from lowercase unigram tokens (regex `[a-z0-9]+`, length > 1) under three stop-word policies:

1. **None** — all tokens retained.
2. **English** — the scikit-learn English stop-word list (the policy used for the

main-text Fig. 3).

3. **Domain** — English plus a curated GO-generic list:

{activity, biological, cell, cellular, dependent, function, involved, mediated, molecular, negative, positive, process, regulated, regulating, regulation, response, system, via}.

Table S4. Biological Process entropy (bits) by depth under each stop-word policy.

Depth	None	English	Domain
**0**	1.00	1.00	—

**1**	4.41	4.14	4.20
**2**	7.75	7.82	8.01
**3**	8.90	9.09	9.35
**4**	**8.96**	**9.22**	**9.75**
**5**	8.47	8.81	9.69
**6**	8.16	8.53	9.56
**7**	7.79	8.14	9.33
**8**	7.43	7.74	8.86
**9**	6.74	7.06	8.04
**10**	5.95	6.13	6.79
**11**	5.04	5.05	5.09
**12**	3.66	3.61	3.34
**13**	3.32	3.19	2.66

Narrative. The **entropy peak is at depth 4 under all three policies**. Removing the generic GO tokens (domain policy) *raises* the absolute entropy at intermediate depths but does not move the peak, confirming that the diversification–specialization profile is **not an artifact of ubiquitous generic tokens**. **Fig. S4** plots the three curves.

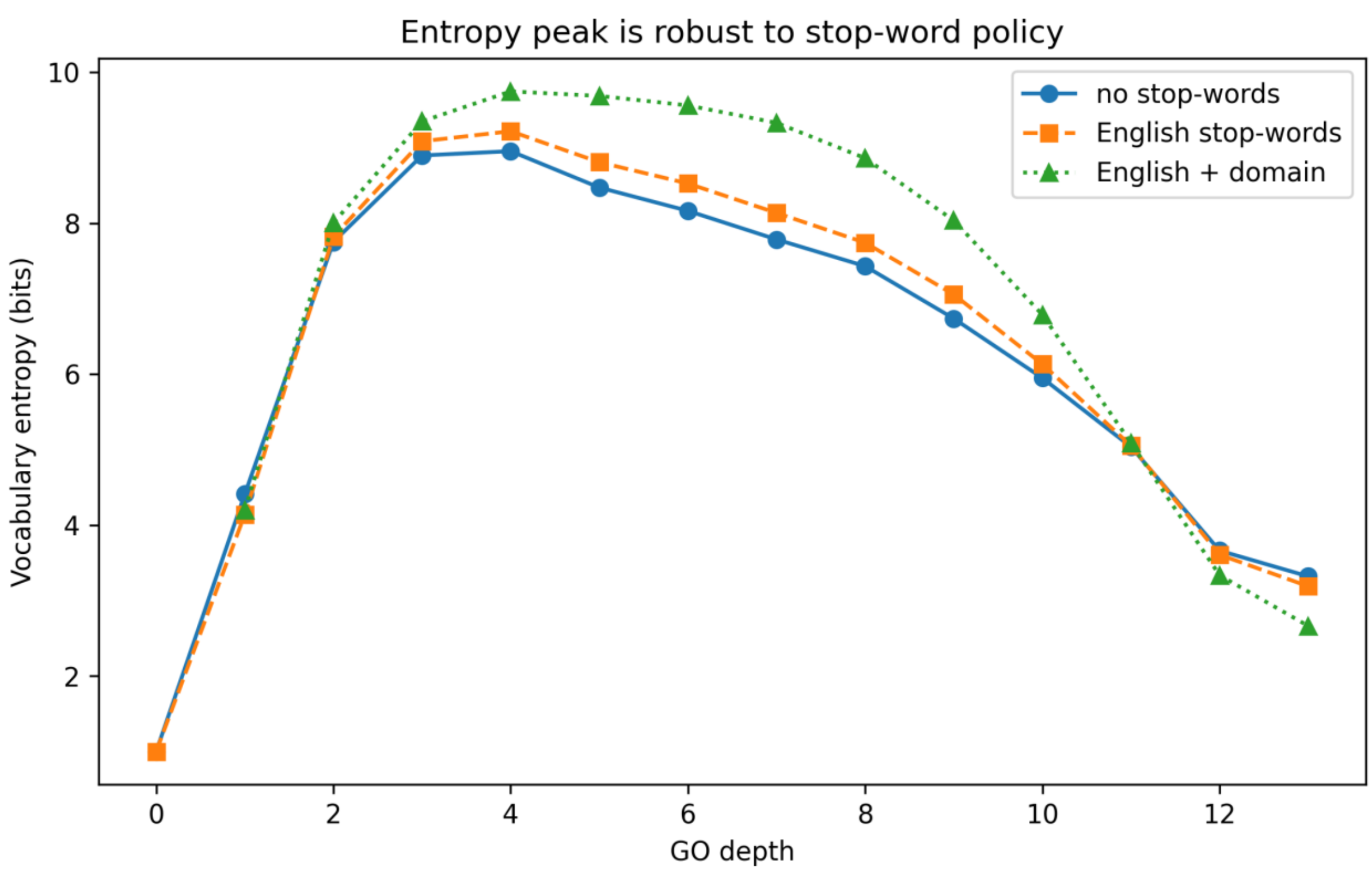

Figure S4. Depth-wise vocabulary entropy under three stop-word policies (none, English, English+domain); the depth-4 peak is preserved.

Alt text: Three vocabulary-entropy curves over depth computed with no stop-words, English stop-words, and English-plus-domain stop-words; all retain the same intermediate-depth peak at depth 4.

S5. Descriptive trend comparison

For illustration only, **Fig. S5** overlays the linear, logarithmic, and quadratic descriptive summaries on the depth-wise Biological Process entropy, drawn with **distinct line styles** (solid / dashed / dotted), contrasting colours, and markers. This overlay was moved here from the main-text Fig. 3 middle panel. **No formal model selection is claimed**: with only ~14 depth levels a three-parameter fit cannot be reliably preferred over simpler forms, so the quadratic is shown purely as a descriptive guide. For reference, its vertex falls at depth ≈ 6.24 (leave-one-depth-out range 5.7–6.4), consistent with — but not used as evidence of — an interior peak. The **empirical** peak, and the substantive evidence for non-monotonicity, are the directly observed maximum at **depth 4** and the Monte-Carlo null envelope (main-text §5.3–§5.4): the observed entropy lies outside the 1–99% null band at depths **1 and 3–13 (12 of 14 depths)**.

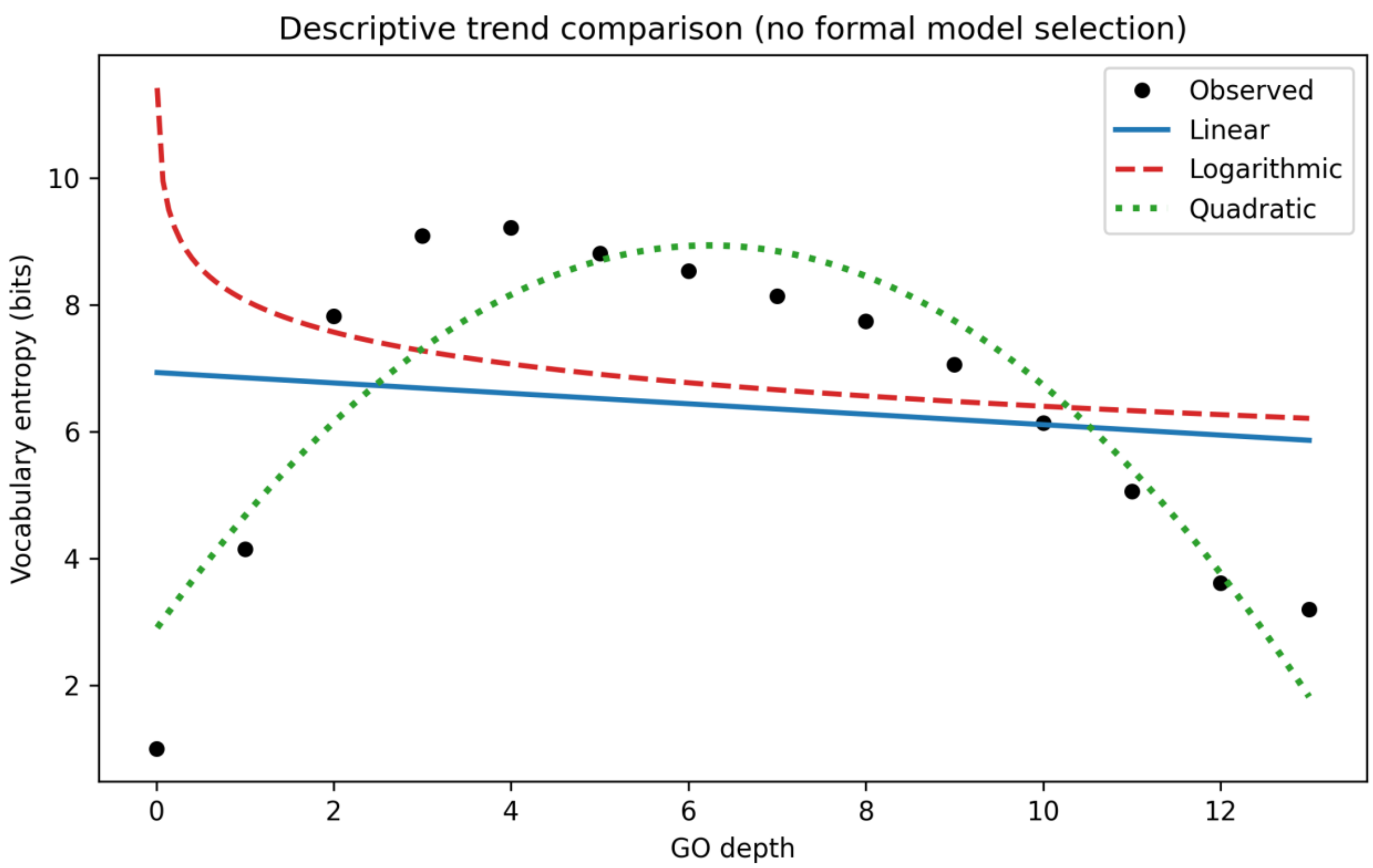

Figure S5. Descriptive linear/logarithmic/quadratic trend overlays on the BP entropy curve (illustration only; no model selection claimed).

Alt text: Observed entropy points with linear (solid), logarithmic (dashed), and quadratic (dotted) descriptive overlays distinguished by style and color; shown for description only.

S6. Reproducibility

All analyses are released as the tested, pip-installable Python package **`go_lingdepth`** together with the figure- and table-generation scripts and the canonical result tables.

- **Ontology input:** `go-basic.obo`, data-version **releases/2026-05-19**, SHA-256

`923645000afcb1df0e72e252b64950b1c2bcc98c9393fec8c1dd1a0f7c9d4d77`.

- **Environment:** pinned in `requirements.txt` (key packages: numpy 2.4.1, pandas 2.3.3,

scikit-learn 1.8.0, scipy 1.17.1, statsmodels 0.14.6, networkx 3.6.1, umap-learn 0.5.12, sentence-transformers 5.6.0, matplotlib 3.10.8, seaborn 0.13.2).

- **Determinism:** random seeds are fixed throughout. Set `PYTHONHASHSEED=0` before

running — Python's string-hash randomization affects `set`-based term ordering, which slightly perturbs the k-means partitions (all remain $p \approx 0$); the committed CSVs are the canonical run. Pinning the seed reproduces the headline $k = 20$ clustering exactly (identical Kruskal–Wallis H across runs) and every order-independent result — correlations, entropy, null models, synthetic correlations, and namespace summaries — bit-for-bit; residual last-digit variation can remain at other k values from BLAS/k-means internals, leaving all conclusions unchanged.

- **Archive:** Zenodo DOI: 10.5281/zenodo.21501473; source mirror

https://github.com/Sadiya-ISU/Letters-and-Biological-Process.
